# Laser Reduced Graphene Oxide Electrode for Pathogenic *Escherichia coli* Detection

**DOI:** 10.1021/acsami.2c20859

**Published:** 2023-02-14

**Authors:** Lei Zhao, Giulio Rosati, Andrew Piper, Cecilia de Carvalho Castro e Silva, Liming Hu, Qiuyue Yang, Flavio Della Pelle, Ruslán R. Alvarez-Diduk, Arben Merkoçi

**Affiliations:** † Catalan Institute of Nanoscience and Nanotechnology (ICN2), Edifici ICN2, Campus UAB, 08193 Bellaterra, Barcelona, Spain; ‡ Department of Chemical Engineering, School of Engineering, Universitat Autònoma de Barcelona, Campus UAB, 08193 Bellaterra, Barcelona, Spain; § MackGraphe-Mackenzie Institute for Research in Graphene and Nanotechnologies, Mackenzie Presbyterian University, Consolação Street 930, 01302-907 São Paulo, Brazil; ∥ Department of Material Science, Universitat Autònoma de Barcelona, Campus UAB, 08193 Bellaterra, Barcelona, Spain; ⊥ Faculty of Bioscience and Technology for Food, Agriculture and Environment, University of Teramo, via Renato Balzarini 1, 64100 Teramo, Italy; # Catalan Institution for Research and Advanced Studies (ICREA), Passeig de Lluís Companys, 23, 08010 Barcelona, Spain

**Keywords:** Graphene electrodes, Fabrication, Biosensing, Nanomaterials, Bacteria detection

## Abstract

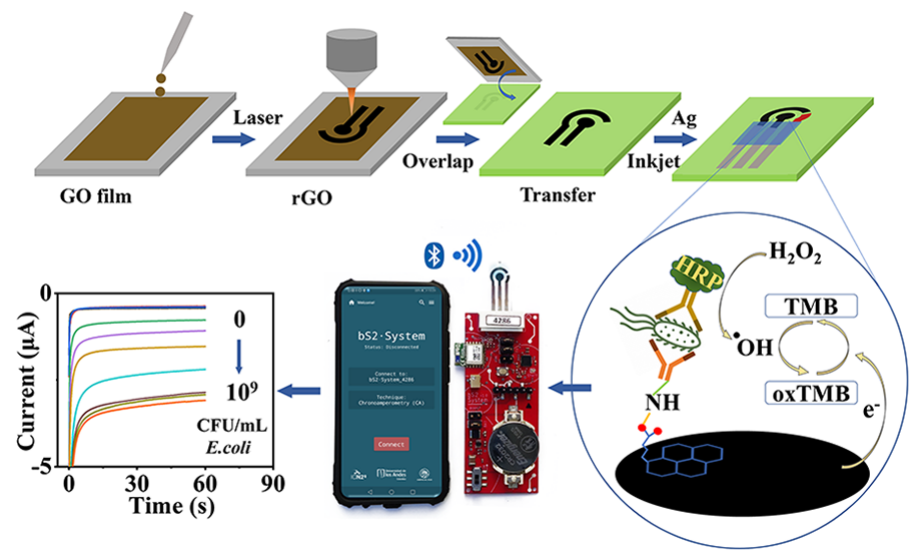

Graphene-based materials are of interest in electrochemical biosensing due to their unique properties, such as high surface areas, unique electrochemical properties, and biocompatibility. However, the scalable production of graphene electrodes remains a challenge; it is typically slow, expensive, and inefficient. Herein, we reported a simple, fast, and maskless method for large-scale, low-cost reduced graphene oxide electrode fabrication; using direct writing (laser scribing and inkjet printing) coupled with a stamp-transferring method. In this process, graphene oxide is simultaneously reduced and patterned with a laser, before being press-stamped onto polyester sheets. The transferred electrodes were characterized by SEM, XPS, Raman, and electrochemical methods. The biosensing utility of the electrodes was demonstrated by developing an electrochemical test for *Escherichia coli.* These biosensors exhibited a wide dynamic range (917–2.1 × 10^7^ CFU/mL) of low limits of detection (283 CFU/mL) using just 5 μL of sample. The test was also verified in spiked artificial urine, and the sensor was integrated into a portable wireless system driven and measured by a smartphone. This work demonstrates the potential to use these biosensors for real-world, point-of-care applications. Hypothetically, the devices are suitable for the detection of other pathogenic bacteria.

## Introduction

Electrochemical biosensors have many inherent advantages over other sensing modalities, including quantitative output, low cost, and high sensitivity.^1−3^ Graphene-based electrodes have shown great potential for developing sensitive electrochemical sensors due to the tunable electrical conductivity, high surface area, versatile functionality, and biocompatibility, with numerous applications in the field of health care, food safety, and environmental monitoring.^4−6^ Low defect graphene is usually produced by physical exfoliation, in which expensive/toxic solvents (*N*-methyl-2-pyrrolidone, dimethyl sulfoxide, and *N*,*N*-dimethylformamide) are used,^6^ or chemical vapor deposition (CVD),^7^ which involves high temperatures and tedious transferring process and has low yields, limiting the real-world applicability of CVD graphene electrodes. To address these issues, “functionalized” graphene,^8−10^ such as graphene oxide (GO), has emerged as an alternative for graphene electrode fabrication. However, the poor conductivity of GO restricts its use in electronic devices.^11^ Reduced graphene oxide (rGO) displays good conductivity and a high electron transfer rate and preserves enough oxygen groups for further functionalization.^12^ Chemical,^13^ thermal,^14^ and electrochemical^15^ methods to reduce GO to rGO have all been reported. Each results in graphene electrodes with different surface compositions and defect densities.

The patterning of graphene electrodes is important for electrochemical sensor fabrication.^4,16,17^ Lithographic techniques are commonly used to pattern low defect graphene;^17,18^ however, the need for expensive equipment and clean room facilities makes these methods inaccessible to most researchers. Template based methods, such as vacuum filtration,^19^ chemical covalent bonding,^20^ and contact transfer printing,^21^ have also been used to pattern graphene, but they require physically or chemically patterned templates, which are laborious to prepare and can require harmful chemicals, limiting their scalability. Printing approaches have been developed to realize the scalable production of low-cost graphene electrodes.^22^ However, in order to make the graphene printable, additives and fillers are often needed to adjust the rheological properties of the inks; this can affect the electrochemical performance of the final printed electrodes. Likewise, the substrates may require chemical modification to have stable and uniform ink adhesion. Additionally, high temperature thermal annealing is often required after printing,^23^ which can affect the material properties of the fabricated devices and limit the choice of substrates to temperature resistant materials. Since printing is a solution-based fabrication process, restacking of graphene flakes is also possible during sintering.^9^ This restacking, driven by van der Waals forces and π–π stacking, reduces the graphene surface area.

One promising method of patterning graphene is with lasers, to create laser-induced graphene (LIG) electrodes.^24−26^ It is an easy, efficient, low-cost, chemical, and mask-free method of simultaneously reducing and patterning GO (or alternatively the in situ creation of graphene). Previous works have been reported by the Kaner group,^27,28^ in which LIG electrodes were fabricated using a standard LightScribe DVD driver; the total cost of this process, including purchasing the laser, was less than $20. Lin et al.^29^ were the first to produce porous graphene via the direct laser scribing of a commercial polyimide sheet with a CO_2_ laser in 2014. Ever since, polyimide substrates have dominated the field, although there has been much research into alternative materials, including other polymers,^16^ carbon, and natural materials (such as wood and leaves) produced by different laser types.^16,30,31^ There have been various works in the literature that focus on not only electrode fabrication but also biosensor development on electrodes formed by laser scribing polyimide films.^16,24,26^ However, at the time of writing, the authors are unaware of any publications showing biosensor development on electrodes created by the laser-induced reduction of GO. The main difficulties that prevent the development of biosensors by this method are as follows. (1) The reduction happens in situ, meaning that there will be GO and rGO in the same plane. Since GO is highly soluble in water, it will dissolve in any biological media.^9,32^ (2) LIG is very fragile, even being damaged by gentle gas flows.^33^ To overcome these issues, the Nam group developed a method of transferring LIG from polyethylene terephthalate (PET), but this method is only applicable to substrates that do not absorb the excitation laser.^34^ The Merkoçi group recently reported a novel, stamp transfer method, capable of fabricating rGO electrodes on a wide variety of substrates.^35^ In this method, a GO film is formed on a polyvinylidene fluoride filter membrane by vacuum filtration, before being simultaneously patterned and reduced by a LightScribe DVD driver. The LIG was then transferred to various substrates with a mechanical press. In this process, the rGO is transferred and the GO is not, while the LIG is compressed, improving its mechanical properties. This proof-of-concept work only verified that the transfer stamped LIG electrodes were capable of electrochemical sensing, not biosensing. In addition, the process required a vacuum pump, could only produce small batch sizes, required expensive filter membranes, and was relatively slow.

Herein we report advancements on our previous work on laser reduced graphene oxide (LRGO) electrode fabrication, to make the production simpler and more scalable, as well as showing their biosensing ability. The LRGO working electrodes were stamped onto polyester (PE) sheets ,and silver inks were inkjet-printed to create silver contacts and Ag/AgCl reference electrodes. A LRGO electrode based biosensor is demonstrated for the first time; as a proof-of-concept, it was developed for the detection of *Escherichia coli* (*E. coli*) by creating an electrochemical enzyme linked immunosorbent assay (ELISA). The sensor was validated in both phosphate buffered saline (PBS) and artificial urine (AU), with a commercial potentiostat and a portable, wireless system driven by a smartphone,^36^ displaying its capability for point-of-care applications.

## Experimental Section

### Materials

GO solution (GO water dispersion, 10 mg/mL) was purchased from Graphenea (San Sebastian, Spain). Polyester (PE) sheets (AUTOSTAT HT1802168201) were obtained from MacDermid Autotype Ltd. (Wantage, UK). Phosphate buffered saline tablets, Tween 20, and Tryptic Soy Agar were ordered from Merck Life Science S.L.U (Madrid, Spain). Phosphate buffered saline buffer (PBS, 10 mM, pH 7.4) was prepared by dissolving PBS tablets in ultrapure water. PBS with Tween 20 (PBST) buffer was prepared by adding Tween 20 (final concentration 0.05 wt %) in PBS buffer (10 mM). Polyclonal anti-*E. coli* antibody (PA1–7213) was obtained from Fisher Scientific (Madrid, Spain). HRP labeled antibodies (ab20425) and 3,3′,5,5′-tetramethylbenzidine (TMB) substrate (ab171523) were purchased from Abcam plc (Cambridge, UK). *Escherichia coli* (*E. coli*, CECT 4972) and *Staphylococcus aureus subsp. Aureus* (SA, CECT 5190) were purchased from CECT (Valencia, Spain), and *Salmonella typhimurium* (ST, ATCC 14028) from ATCC (Virginia, USA).

### LRGO Electrode Production

PE substrates were washed with isopropanol (IPA) and dried with nitrogen or compressed air. Afterward, 5 mL of GO solution was drop-casted on an 8 × 8 cm^2^ square defined on the PE sheet with paper tape (to prevent the solution from spreading), shaken gently by hand, and then dried at 60 °C for 2 h. A CO_2_ laser engraver (Rayjet 50 Laser Engraver, Trotec Laser GmbH, Marchtrenk, Austria) was used to scribe the GO film to produce laser reduced graphene oxide (LRGO). The laser settings were as follows: power = 2.07 W and speed = 1.5 m/s. The LRGO was placed face down on the desired substrate, fixed tightly in place with scotch tape, sandwiched between four sheets of A4 paper (2 layers above and 2 layers below the sample), and placed in a hydraulic press (SPECAC manual hydraulic press 15 ton, UK). A 6-ton force was applied for 1 min to transfer the LRGO onto the hosting substrate, forming both the working electrode (WE) and counter electrode (CE). Silver contacts were inkjet printed with a Dimatix 2831 (FUJIFILM Dimatix, USA. Print temperature 30 °C, plate temperature 60 °C, drop space 25 μm, 3 layers), using a silver ink (Sicrys I40DM-106, PV Nano Cell Ltd., Migdal Ha’Emek, Israel) and then oven-cured at 160 °C for 2 h. Polydimethylsiloxane (PDMS, SYLGARD 184 silicon elastomer kit, Dow corning, MI, USA) was used as an insulating layer; a schematic summary of the insulation process has been included in Figure S1. Ag/AgCl pseudoreference electrodes (RE) were obtained by chlorinating the printed Ag trace with 0.4% NaClO for 3 min.^37^

### Characterizations of LRGO

Cyclic voltammetry (CV) was done with a commercial potentiostat (PalmSens4, PalmSens BV, Houten, Netherlands), in an aqueous solution of 0.5 mM potassium ferrocyanide, 0.5 mM potassium ferricyanide, and 0.1 M KCl at scan rate of 50 mV/s. For defining the electrochemical active surface area (ECSA), CVs were performed in 0.1 M NaClO_4_ (in ethanol) by using a 3 mm diameter LRGO disk as the WE, four LRGO squares (Each 1 cm^2^) as CEs, and a commercial Ag/AgCl electrode (CHI111, CH Instruments, Inc.) as the RE. The height of the films was measured by an optical profilometer (Profilm 3D, Filmetrics Inc.) equipped with a 10× objective and using a stage movement speed of 0.12 mm/s. Scanning electron microscopy (SEM) was performed with a Quanta 650 (UK) at a working distance of 10 mm and voltage 10 kV; the samples were mounted on aluminum stubs and sputtered with Au prior to imaging. The sheet resistance was obtained by measuring square (1 × 1 cm^2^) samples with a Keithley 2400 using a four-point probe system (van der Pauw method). Raman spectra were obtained with a Raman microscope (Alpha300 R-Raman Imaging Microscope, Oxford Instruments) using a 488 nm laser with a spot size of approximately 3 μm. The parameters used were as follows: laser power = 1 mW, grating = 600 gr/nm, objective = 50×, exposure time = 10 s, accumulations = 2, waveform range = 500–3500/cm. X-ray photoelectron spectroscopy (XPS) measurements were performed at room temperature with a SPECS PHOIBOS 150 hemispherical analyzer (SPECS GmbH, Berlin, Germany) at a base pressure of 5 × 10^–10^ mbar using monochromatic Al Kα radiation (1486.74 eV) as the excitation source, operated at 300 W. The energy resolution as measured by the full width at half-maximum (fwhm) of the Ag 3d 5/2 peak for a sputtered silver foil, and was found to be 0.62 eV. The fitting of the obtained spectra was done with XPSPEAK 41 with a Shirley background correction.

### LRGO Electrode Functionalization

The LRGO working electrode was activated with 1-pyrenebutanoic acid succinimidyl ester solution (PBASE, 1 μL of 2 mg/mL in dimethylformamide) at room temperature for 4 h. The electrode was then cleaned twice with IPA and four times with ultrapure water. The capture antibody (cAb, 15 μg/mL in 10 mM PBS) was spontaneously immobilized by incubating 5 μL of the solution on the WE (4 °C, overnight). Ethylamine (4 μL, 0.1 M in PBS) was put on the WE for 30 min to block any unreacted PBASE, and then the electrode was further blocked with bovine serum albumin (BSA, 5 μL, 5% in PBS) at 37 °C for 1 h to prevent nonspecific fouling in complex media. Between each step, the electrode was washed twice with PBST and twice with PBS; all the steps above were performed in a humidity chamber.

### Bacteria Preparation

Bacteria were cultured overnight in Tryptic Soy Agar at 37 °C. Afterward, colonies were picked and suspended in PBS. Then the bacterial solutions were diluted to obtain an OD600 value as close to 10^9^ CFU/mL as possible.^38^ Finally, the bacteria were heat-killed at 65 °C for 20 min.^39^

### Electrochemical Bacterial Detection

To perform the bacteria detection, 5 μL of the bacteria-containing solution was pipetted onto the WE, left for 30 min, and then washed twice with PBST and twice with PBS. Directly after cleaning, 4 μL of the detection antibody solution (dAb, ab20425, 2 μg/mL) was pipetted onto the WE and left for 30 min, and then washed twice with PBST and 4 times with PBS. Subsequently, 50 μL of the TMB ELISA solution was added, and the chronoamperometric sensing started. Chronoamperometry (CA) was performed at +0.125 V, vs the on-board Ag/AgCl RE, with both the PalmSens4 and a wireless smartphone driven potentiostat developed in house and fabricated according to a previous publication.^36^

### ELISA Bacterial Detection

The ELISA test was performed according to a previously established protocol with minor modifications.^40^ First, 100 μL of capture antibody (PA1–7213, 2 μg/mL in Carbonate-Bicarbonate Buffer, pH 9.6) was aliquoted into a 96-well ELISA plate (44–2404–21, MaxiSorp, Fisher Scientific, Spain) and incubated at 4 °C overnight. Second, 200 μL of BSA (3% in PBS) was added into the wells and incubated at 37 °C for 1 h, to prevent nonspecific fouling of the wells. Third, 100 μL of bacterial samples was added and kept at room temperature for 1h. The wells were washed three times with PBST between each step, and then incubated with 100 μL of HRP-labeled detection antibodies (HRP-dAb, 0.33 μg/mL ab20425, in PBS) for 1 h at room temperature. Finally, the ELISA wells were washed 5 times with PBST. Afterward, 100 μL of the TMB substrate solution was added into each well and left for 10 min at room temperature. Finally, 50 μL of 2 M H_2_SO_4_ was added into the wells and then the absorbance of each well was detected at 450 nm using a SpectraMax iD3Multi-Mode Microplate Reader (Molecular Devices, USA).

## Results and Discussion

**Figure 1:**
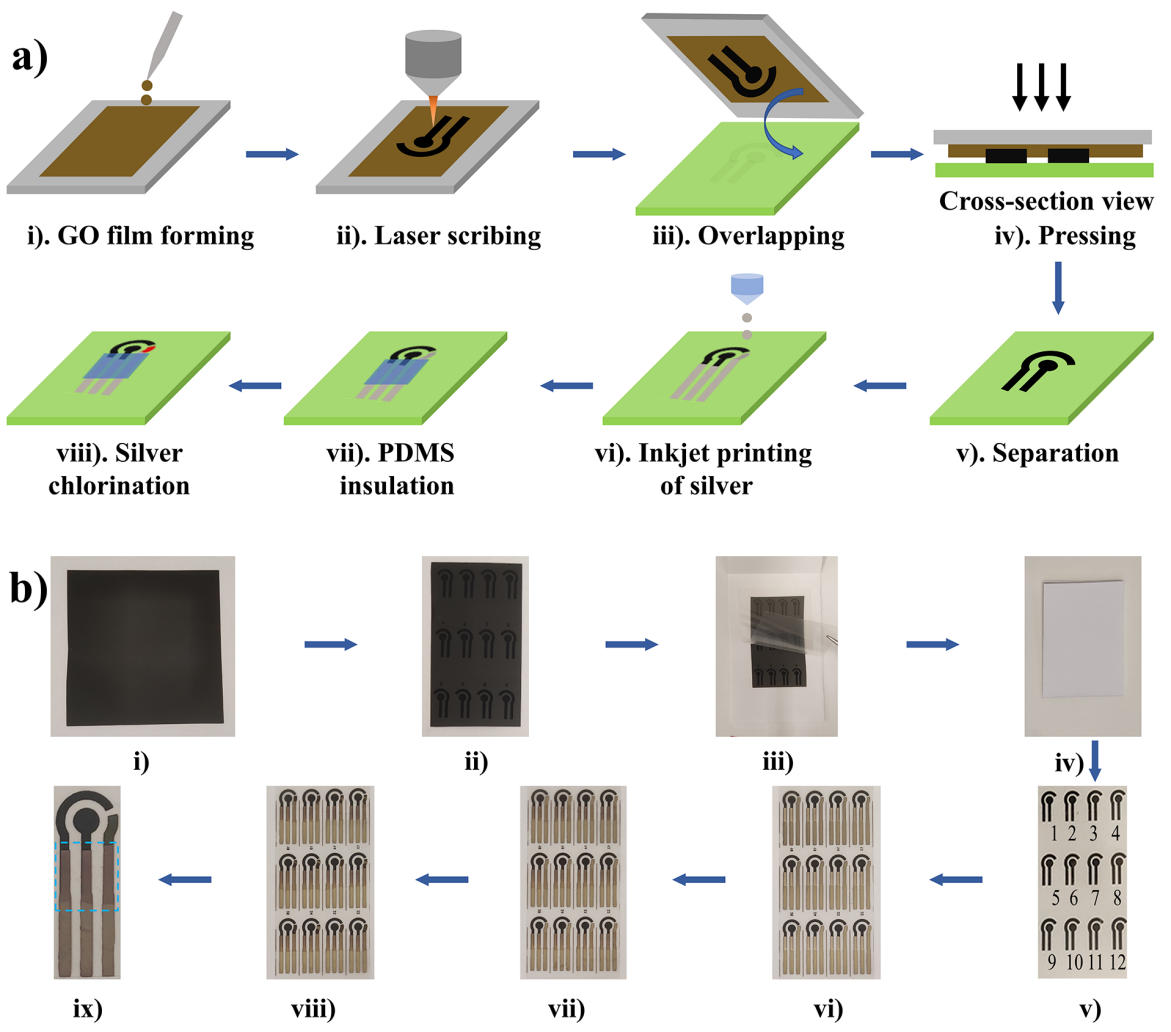
(a) Schematic of the laser reduced graphene oxide (LRGO) electrode fabrication process. (i) A graphene oxide (GO) film was formed on a polyester (PE) sheet by drop casting GO solution in a defined area and drying at 60 °C; (ii) laser scribing to reduce and pattern the GO film, creating LRGO; (iii) LRGO was placed face down on the desired substrate; (iv) pressure was applied to stamp the LRGO; (v) after separation, a mirror image of the LRGO had been transferred; (vi) inkjet printing of silver connections; (vii) polydimethylsiloxane (PDMS) was cast as a dielectric layer; (vii) NaClO was used to convert Ag to Ag/AgCl as reference electrode. (b) Photographs corresponding to each of the steps shown in (a); (ix) magnification of the final electrode, consisting of LRGO working electrode (black dot), LRGO counter electrode (black arc) and Ag/AgCl pseudoreference electrode (dark brown arc) and PDMS insulation layer inside the dashed square.

**Figure 2:**
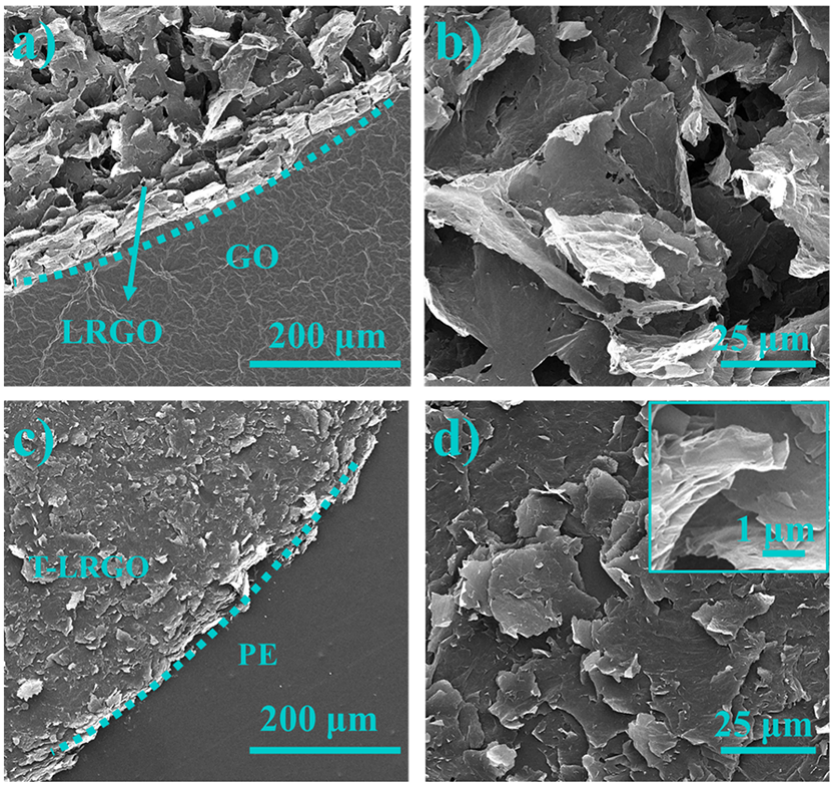
(a) SEM image of the boundary between the LRGO and GO film, showing the morphological and topographical changes in the graphene upon reduction. (b) Magnified SEM image of the LRGO showing the flake-like structure. (c) SEM image of the LRGO after transferring onto a polyester (PE) sheet, dubbed T-LRGO. (d) Magnified image of the compressed flake-like structure of T-LRGO on PE; inset is a high magnification of the same region to evidence the flake structures.

In this work, all the digital designs were created with AutoCAD 2018 (Autodesk, USA) and then sent to the laser engraver/inkjet printer to produce the patterns, this direct writing strategy along with the stamp-transferring method enables the simple, fast, maskless and large-scale production of LRGO electrodes (Figure 1). SEM image of the GO film surface (Figure 2a), shows that the surface has the bumps and ravines typical of graphene-based materials.^9,32,41^ After laser treatment, the film becomes a foam-like, porous structure, composed of flakes 10s of microns long (Figure 2a and b). Additionally, the GO changed from a brown color to black (Figure 1, i, ii) upon reduction, in agreement with the literature.^35^ Although the LRGO surface is smoother and less porous after the stamp-transferring process, it still exhibits a highly porous 3D framework (Figure 2c and d). The data from the optical profilometer measurements, Figure S2, show that the GO film is 3–4.5 μm thick; after laser irradiation, the surface becomes much more heterogeneous but noticeably increases to a maximum height of 18.2 μm in some areas. The laser scribing of GO is a photothermal process; although the exposure time is only microseconds, the instantaneous temperature at the surface is over 1000 °C,^16^ which causes the surface to rapidly expanded and blister, this is proposed as the root cause for the changes in surface morphology in these experiments. A large pressure is applied to the film so it is pressed flat during transferring, hence the decrease in thickness to 0.4–1.3 μm.

**Figure 3:**
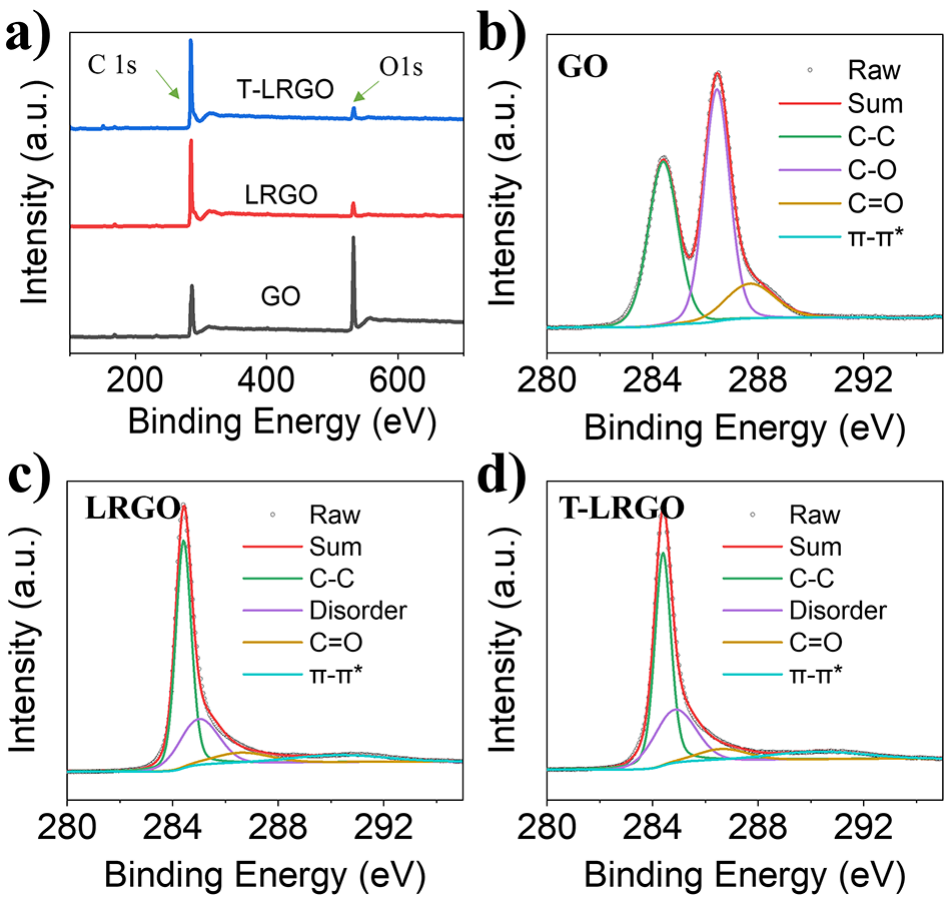
(a) XPS survey and high-resolution C 1s spectra of (b) GO, (c) LRGO, and (d) T-LRGO. The survey spectra show a significant decrease in O content after laser radiation; C 1s spectra of GO reveals an abundance of oxidized carbon C–O (286.4 eV) and C=O (287.7 eV); the dominance of the C–C (284.4 eV) bond and presence of π–π* (291.2 eV) in LRGO and T-LRGO proves GO has been reduced by the laser.

GO, LRGO and T-LRGO were characterized via XPS, in all these samples the C and O peaks dominated (Figure 3a). The increase in the C/O ratio when the GO (2.7) is reduced to LRGO (22.0) is expected and confirms that the laser reduction was successful. During the stamp-transferring step, the surface of the LRGO is placed in contact with the new substrate and the surface of the T-LRGO is, what was once, the bulk of the LRGO. Therefore, the similar C/O ratio between T-LRGO (23.4) and LRGO (22.0) indicates that the laser-induced reduction is not a surface confined process but penetrates into the bulk of the GO. The high-resolution C 1s spectra of GO (Figure 3b) display peaks at 284.4, 286.4, and 287.7 eV, which can be assigned to sp^2^ hybridized carbon in the graphene lattice (C–C), sp^3^ carbons in the form of epoxide and hydroxyl carbons (C–O) and sp^3^ carbons in the form of carbonyl groups (C=O), respectively.^28,42,43^ Further analysis shows the relative peak areas are 39.0% (284.4 eV), 46.8% (286.4 eV), and 13.9% (287.7 eV), Table S1; the high proportion of oxygen containing functional groups shows the degree of oxidation of the GO. In the C 1s spectra of LRGO (Figure 3c), the presence of a disordered carbon peak at 285.0 eV (25.6%)^28,43^ with a small C–O peak (8.8%) and the disappearance of the C=O peak further proves the effective reduction of the GO by the laser scribing. The increase in sp^2^ hybridized carbon content (C–C, 56.4%) and significant increase in the π–π* (at 291.2 eV, 9.2%; and 0.3% in the case of GO) satellite peak shows that the graphene lattice has been restored upon reduction of the GO. The data from the LRGO and T-LRGO samples are the same within error, showing that the reduction is uniform to the transferred graphene depth and that the transferring has no effect on the chemistry of the graphene. The degree of reduction GO reduction is clearly very high for this process, given all the above, but was further proven by the improved conductivity of the materials, the GO had a sheet resistance of >20 MΩ/sq,^27^ which decreased to 90.6 ± 3.9 Ω/sq for LRGO (the thickness was assumed to be 1 μm).

**Figure 4:**
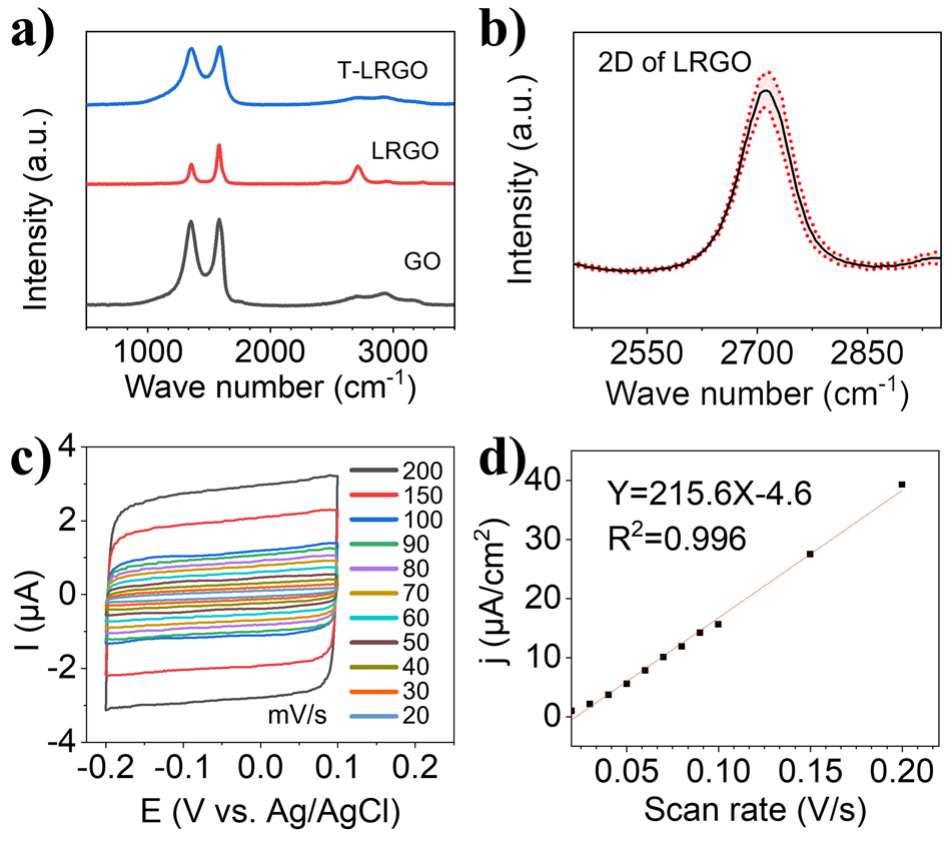
(a) Representative Raman spectra of GO, LRGO, and T-LRGO on PE, showing the typical D (∼1345 cm^–1^) and G (∼1580 cm^–1^) bands of graphene; the 2D band and decreased *I*_D_/*I*_G_ ratio in LRGO are indicative of GO reduction by laser scribing. (b) Average 2D band from 5 measurements of LRGO (shaded area refers to the standard deviation of the average), showing that the LRGO contains only a few graphene layers, i.e., is not multilayered graphene. (c) Cyclic voltammograms obtained in a solution of 0.1 M NaClO_4_ in ethanol vs a LRGO CE (4 cm^2^) and a Ag wire pseudoreference electrode, at a series of scan rates shown in the inset. (d) Current density from (c) plotted as a function of the scan rate, to determine the electrochemical active surface area (ECSA) of the electrodes. The black dots represent raw data and red line is a linear fit to the data.

The Raman spectroscopy data (Figure 4a) shows that the characteristic D (∼1345 cm^–1^) and G (∼1580 cm^–1^) bands of graphene are present in all three samples, with intensity ratios (*I*_D_/*I*_G_) of 1.00 ± 0.02 for GO, 0.41 ± 0.07 for LRGO and 0.99 ± 0.01 for T-LRGO (*n* = 6). The 2D band, at ∼2700 cm^–1^, was only present in the LRGO samples. Previous publications^41,44,45^ have assigned the D peak to the primary in plane vibrations of graphene, so it can be used to estimate the degree of defects and disorder within the hexagonal lattice. The 2D peak is attributed to second-order in-plane vibrations of graphene. The significant decrease of *I*_D_/*I*_G_ after laser exposure is therefore indicative of rGO formation, which is in accord with the XPS and sheet resistance findings. The normalized average 2D peaks from 5 LRGO spectra recorded at random locations on a single working electrode surface, Figure 4b, show small standard deviations; this indicates a homogeneous reduction of GO across the electrode surface. The full width at half maximum (FWHM) of the 2D bands were extracted using the Lorentzian function (Figure 4b), for the LRGO the FWHM was found to be 74.97 ± 2.93 cm^–1^, indicating that the LRGO film consists of only a few graphene layers.^28,46,47^ The Raman spectra of the T-LRGO are the same within error as that of the GO. It is proposed that there are residual amounts of unreduced GO on the surface of the transferred electrodes. Raman spectroscopy is a surface measurement so the GO lying on the surface of the electrode dominates the signal. This was not observed in the XPS since it has much larger sampling area (spot size 100s μm, and 3 μm in the case of Raman) and probes the chemistry up to a certain depth (depending on the substrate and experimental conditions), reporting the average chemical composition of a volume at the surface. We can therefore surmise that there is a thin layer of GO on the surface of the T-LRGO.

The surface area, roughness, and porosity of electrodes can directly influence the performance of electrochemical sensors.^1,25^ To ascertain the electrochemically active surface area (ECSA) of the LRGO electrodes, non-Faradaic CV was performed in 0.1 M NaClO_4_ in ethanol. The CVs of the electrodes at scan rates from 20 to 200 mV/s are provided in Figure 4c. Plotting current density (*j*) against the scan rate (υ) (Figure 4d), a linear relationship was obtained, in which the slope of the linear plot is equal to the area specific double layer capacitance (*C*_S_), and the *C*_S_ of the electrode is directly related to its ECSA through eqs 1–3:^48^
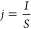
1
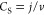
2
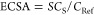
3where *S* is the geometric surface area of the electrode; *υ* is scan rate; *I* is the current; *j* is current density; *C*_ref_ is the area specific double-layer capacitance of a flat reference surface.

Equationss 1–3 and the data in Figures 4c,d and S3 were used to calculate the ECSA of the electrodes based on the C_ref_ of graphite (∼20 μF/cm^2^);^49,50^ the average electrode area from three repeats was found to be 0.781 ± 0.022 cm^2^. The ECSA is more than 10 times larger than the geometric area (0.07 cm^2^), which is expected given the highly rough and porous nature of the electrodes. The standard deviation of the three repeats speaks to the reproducibility of the LRGO working electrodes.

Inkjet-printed silver was used to create electrode contacts and to fabricate the reference electrode.^37^ Open circuit potential measurements of the printed quasi-reference electrodes (*N* = 4) vs a commercial Ag/AgCl electrode (CHI111, CH Instruments, Inc.) exhibit potential fluctuations of less than 4 mV over 200 s, and a potential difference of less than 14 mV (Figure S4a), verifying the functionality of the prepared reference electrodes.

Figure 1b, ix shows a photograph of the final electrodes, consisting of LRGO working and counter electrodes, and Ag/AgCl quasi-reference electrode. The electrodes are made in batches; it is therefore prudent to assess the reproducibility of the electrochemical signal obtained from multiple batches. To do this, 3 batches of electrodes were fabricated and 6 electrodes from each batch selected at random for testing (a total of 18 electrodes were used). These were first modified with PBASE to make them hydrophilic, and CVs were recorded in 0.5 mM [Fe(CN)_6_]^4–^/[Fe(CN)_6_]^3–^ with 0.1 M KCl. The peak potentials were identical for all the electrodes tested, which further shows the reproducibility of the quasi-reference electrode fabrication (Figure S4b). The relative standard deviation (RSD) of the anodic peak current shows the intrabatch variation is 4.2%, and the interbatch variation is 5.6%.

To prove that the electrodes have the potential to be used for biosensor development, an immunoassay for the detection of pathogenic bacteria was developed on them. *E. coli* was used as a model bacteria in these proof-of-concept tests, since their detection is important for medical, food safety, and environmental monitoring.^39,51^ First a conventional ELISA was run to select the best capture and detection antibody pairs and act as a reference with which the performance of the T-LRGO electrodes could be compared. The control ELISA was capable of detecting *E. coli* between 9.35 × 10^4^–9.06 × 10^6^ CFU/mL and had a limit of detection (LOD) of 1.30 × 10^4^ CFU/mL (Figure S5a).

**Figure 5:**
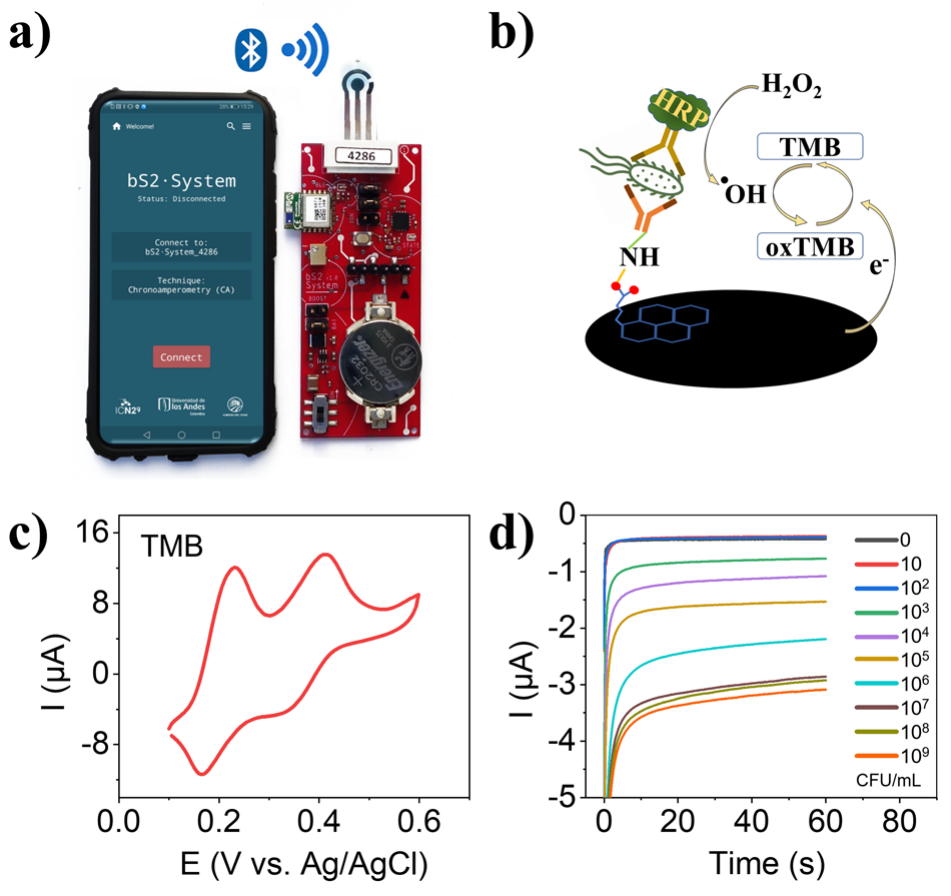
(a) Smartphone controlled portable wireless system for *E. coli* detection (not to scale). (b) Illustration showing the working mechanism of the electrochemical enzyme linked immunosorbent assay (ELISA). (c) Cyclic voltammograms of TMB on the functionalized T-LRGO electrode. (d) Representative current vs time transients of various *E. coli* concentrations, recorded at +0.125 V vs Ag/AgCl.

The same antibodies used in the conventional ELISA were then used to functionalize the T-LRGO and optimized to create a point of care biosensing system, that could be wirelessly driven by a smartphone, Figure 5a (see electric scheme in Scheme S1).^36^Figure 5b shows a schematic representation of the electrochemical sensing mechanism. In brief, HRP catalyzes the generation of OH radicals from H_2_O_2_, and these radicals oxidize TMB. The oxidized TMB (oxTMB) can be detected optically or electrochemically. When the oxTMB is reduced, the reductive current generated is proportional to the amount of oxTMB present. Given that the amount of oxTMB is determined by the amount of dAb, which is proportional to the amount of *E. coli* bound to the electrode surface, the reductive current gives a quantitative evaluation of the amount of *E. coli* in the sample. In other words, the amount of oxTMB is a directly proportional of the amount of *E. coli* bound to the electrode.

**Figure 6:**
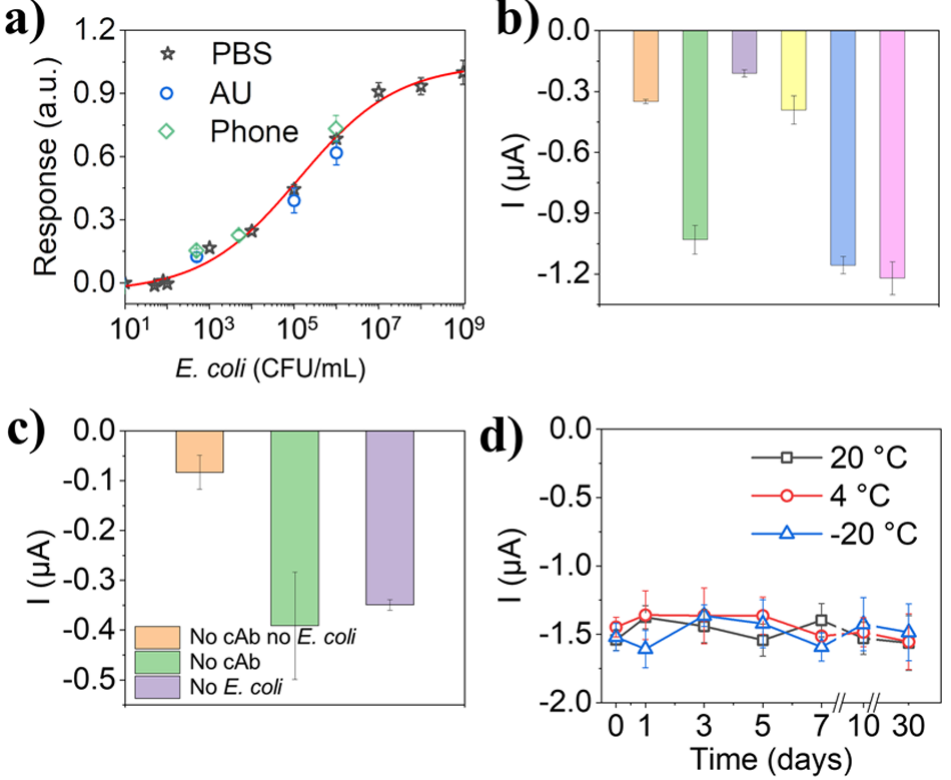
(a) Calibration curve of *E. coli* concentration vs normalized current response from the chronoamperometry at +0.125 V; the black stars were recorded with a commercial potentiostat in PBS, the green diamonds were recorded with the smartphone driven potentiostat developed in house, and the blue circles were obtained with a commercial potentiostat in artificial urine (AU). (b) Selectivity studies showing the current response without bacteria (orange), with *Escherichia coli* (green), *Staphylococcus aureus* (purple), *Salmonella typhimurium* (yellow), *Escherichia coli* and *Staphylococcus aureus* (blue), and *Escherichia coli* and *Salmonella typhimurium* (pink). In all cases, each bacterial concentration was 10^4^ CFU/mL, with a 50:50 mix of each in the last two conditions. (c) Control experiments run either without the cAb, *E. coli* or without both. (d) Data from the stability studies of the sensors stored in different conditions for 1 month, measuring the current response to 10^5^ CFU/mL of *E. coli*.

Figure 5c displays a CV of TMB on the functionalized LRGO electrode. Oxidation peaks at +0.231 and +0.411 V and reduction peaks at +0.166 and +0.367 V can all be easily distinguished (all potentials vs the on-chip Ag/AgCl electrodes). The current time transients obtained after incubating the functionalized electrodes with *E. coli* concentrations between 0 and 10^9^ CFU/mL are provided in Figure 5d. As expected, the normalized dose–response curve in Figure 6a shows a sigmoidal response. With the 4-Parameter Logistic model fitting^52,53^ the LOD was calculated to be 283 CFU/mL (blank + 3SD) with a detection range between 917–2.1 × 10^7^ CFU/mL (lowest and highest concentrations of the analytes based on 10% and 90% of the maximum signal respond on the calibration curve). The improved sensing performance of the LRGO electrode based platform compared with the standard optical ELISA(Table S2), is in accord with other reported electrochemical ELISA platforms.^52^ The performance of the sensor is suitable for the detection of *E. coli* in urine at clinically relevant concentrations (10^3^ – 10^5^ CFU/mL)^54,55^ and it is advantageous that all the results were obtained from 5 μL samples. Ultrasensitive detection, even down to single cell, has been demonstrated,^56−58^ but tedious material synthesis and complicated sensor construction are typically required (Table 1). Our platform showed comparable performance with other electrochemical sensors,^59−61^ and could be improved with further optimization of the antibody concentrations (this work is mainly focused on the fabrication and characterization of the electrodes, with the sensor construction being merely a proof-of-concept).

**Table 1:** Representative Nanomaterial-Based Biosensors for *E. coli* Detection Reported in Literature^a^

platform	technique	detection range (CFU/mL)	LOD (CFU/mL)	response time (min)	sample volume (μL)	ref
metal NPs-LIG	EIS	1 × 10^2^–1 × 10^8^	10^2^	30		(59)
silica NPs	CV	8 × 10^4^–8 × 10^6^	2 × 10^3^	30	10	(61)
Au NPs	LFA	10^4^–10^6^	10^4^	10	150	(39)
Fe_3_O_4_@SiO_2_/ polymer/fluorescein	fluorescent	4–4 × 10^8^	3	75	1000	(56)
MWCNTs/chitosan/thionine	CV	10^2^–10^9^	10^2^	180	200	(60)
N-GQDs/MIP	ECL	10–10^7^	5	140		(57)
rGO/Al_2_O_3_/Au NPs	FET	1–100	single cell	50 s	1	(58)
MoS_2_/Au/optical fiber	SPR	10^3^–8 × 10^9^	94	∼15		(62)
LRGO	CA	917–2.1 × 10^7^	283	60	5	this work

a NPs = nanoparticles; N-GQDs = nitrogen dopped graphene quantum dots; MWCNTs = multiwall carbon nanotubes.

To test the selectivity of the sensors, *Salmonella enterica serovar Typhimurium* (ST) and *Staphylococcus aureus subsp. Aureus* (SA) were run in place of and alongside *E. coli*. The results of these tests are given in Figure 6b, where it can be seen that neither of these bacteria gave a signal significantly above that of the blank. Likewise, when run alongside *E. coli*, the responses are the same within error as those of *E. coli* alone. The same trends were seen in the ELISA tests, Figure S5b; proving that the sensor is specific and that specificity is attributable to the antibodies. Other control experiments, Figure 6c, showed that the signal response is negligible (−0.083 ± 0.034 μA, *N* = 4) in the absence of *E. coli* and cAb; it increases to −0.349 ± 0.010 μA when only *E. coli* is excluded (blank control, *N* = 4), indicating that there is some cross-reaction between the antibodies, which is common when working with polyclonal antibodies.^63,64^ When only the cAb was omitted, the current (−0.391 ± 0.108 μA, *N* = 4) was the same within error as the blank control, indicating insignificant nonspecific binding of *E. coli* to the electrodes.

The stability of the sensors was tested in different storage conditions. After functionalization, batches of the sensors were stored in humidity chambers placed at room temperature (taken as 20 °C), in the fridge (4 °C) and in a freezer (−20 °C), all under N_2_. The *E. coli* detection was performed at several time points over 30 days, Figure 6d (*N* = 4). The signal variability at 20 and 4 °C was found to be similar (RSD ≤ 7.6%) within the first 10 days, comparable to that of the variation in the electrodes calculated earlier (5.6%); even stored for 30 days, the sensors showed similar performance in all cases (RSD of the current response, 12.8% for 4 °C, 13.0% for 20 °C, 14.0% for −20 °C); these data show that the sensors are relatively stable for 30 days and the storage temperature makes no significant difference to the sensor stability. The slightly higher RSD for the sensors stored at −20 °C may come from the freeze–thawing cycles when taking them in and out of the freezer, which could damage the antibodies.^65^

The efficacy of the sensors was further evaluated in spiked artificial urine. The results of these tests are shown in Figure 6a (the blue circles) and correlate very well with those obtained in PBS. The recovery rates obtained are 119% for 500 CFU/mL, 88% for 10^5^ CFU/mL, and 90% for 10^6^ CFU/mL of *E. coli*, showing that the system has the potential to be used in real world matrices.

In order to create a point-of-care (POC) test, there is a need for sensitive, inexpensive, wireless, portable detection platforms.^6,66−68^ A previously established smartphone-based wireless system^36^ was used to make this test more suitable for POC applications. Three concentrations of *E. coli* in PBS were tested with said wireless system and compared to a commercial potentiostat. Movie S1 shows the real-time response of the system. The current responses obtained for three *E. coli* concentrations are the same within error as those recorded with a commercial potentiostat (Figure 6a, green diamonds).

The scalability of the electrode fabrication has been proven by the production of ∼500 electrodes in 1 day, although the whole fabrication process for a single batch of 12 electrodes takes ∼4.5 h. In fact, more than 2000 electrodes could be made under ideal conditions, as calculated from Table S3. It is also worth mentioning that the cost of GO is estimated at € 0.06 per electrode, 4 times cheaper than polyimide. The total materials cost per electrode is € 0.09, and the whole sensor costs less than € 0.12 (Table S4). While the CO_2_ lasers used herein are not common laboratory equipment, there are other low-cost laser sources with wavelengths ranging from 248 nm to 10.6 μm that can be used for LRGO formation.^16,25,27,35,69^ The printing of Ag connections and the reference electrodes can also be realized by much cheaper methods, such as consumer inkjet printers, screen/stencil printing, or simply painting silver inks. These features mean that T-LRGO based sensing platforms can be produced in most research laboratories around the world.

## Conclusions and Future Work

In this work, we reported a cheap and scalable method of fabricating LRGO-based electrodes on polyester substrates. The biosensing potential of the electrodes was proven by functionalizing them for the detection of *E. coli* in PBS and artificial urine. This proof-of-concept sensing platform could detect *E. coli* between 917 and 2.1 × 10^7^ CFU/mL, with a LOD of 283 CFU/mL, within the clinically relevant range for *E. coli* in human urine. We believe that the platform can be utilized for the detection of other bacteria or biomarkers, simply by changing the antibodies.

Being able to transfer these graphene-based sensors onto different substrates would be of interest to other sensing applications, such as wearable sensing.^6,36,44^ An initial study showed that these LRGO films could be transferred onto a variety of other substrates, Figure S6, demonstrating the potential applicability of these electrodes. It is also of note that the maximum resolution of the laser will define the resolution with which electrodes can be patterned. With our equipment this was down to the micron scale, making microelectrode development with this technology possible. Future work will include the development of microelectrode arrays by this method and the optimization of printing and stamping onto other substrates for different applications.

## Supporting Information Available

Illustration of PDMS insulation process; one-dimensional surface profile; XPS C 1s elemental composition; current density at different scan rates obtained with 0.1 M NaClO4 in ethanol of two T-LRGO electrodes; open circuit potential measurements of the as-prepared pseudoreference electrodes vs a commercial Ag/AgCl electrode, CV measurements of PBASE functionalized electrodes; ELISA calibration curve of *E. coli* and selectivity test by ELISA; 4-Parameter Logistic model fitting parameters for the stander optical ELISA and as-prepared electrochemical ELISA; the timetable of electrode fabrication in detail; detailed material costs for the device fabrication; electric scheme of the wireless electrochemical measurement unit; T-LRGO patterns on different substrates (PDF)
Real-time response of the portable wireless system (MP4)

## Abbreviations

GO: graphene oxide
rGO: reduced graphene oxide
LIG: laser-induced graphene
*E. coli*: *Escherichia coli*
LRGO: laser reduced graphene oxide
T-LRGO: transferred laser reduced graphene oxide
PDMS: Poly dimethylsiloxane
WE: working electrode
CE: counter electrode
RE: reference electrode
PBASE: 1-pyrenebutanoic acid succinimidyl ester solution
cAb: capture antibody
dAb: detection antibody
LOD: limit of detection
CV: cyclic voltammetry
CA: chronoamperometry
SA: *Staphylococcus aureus subsp. Aureus*
ST: *Salmonella typhimurium*
XPS: X-ray photoelectron spectroscopy
SEM: scanning electron microscope
AU: artificial urine
