# Supplementary material for: Laser Reduced Graphene Oxide Electrode for Pathogenic Escherichia coli Detection
Source: ACS Appl Mater Interfaces. 2023 Feb 14;15(7):9024–33. doi: 10.1021/acsami.2c20859 (PMC9951213; doi:10.1021/acsami.2c20859)
Supplement: Supplementary file 1 — am2c20859_si_001.pdf [file am2c20859_si_001.pdf]

Supplementary Information

Laser Reduced Graphene Oxide Electrode for Pathogenic *Escherichia coli* Detection

Lei Zhao [a,b], Giulio Rosati *[a], Andrew Piper *[a], Cecilia de Carvalho Castro e Silva [c], Liming Hu [a,b], Qiuyue Yang [a,d], Flavio Della Pelle [e], Ruslán R. Alvarez-Diduk [a], Arben Merkoçi *[a,f]

[a] *Catalan Institute of Nanoscience and Nanotechnology (ICN2), Edifici ICN2, Campus UAB, 08193, Bellaterra, Barcelona, Spain*

b Department of Chemical Engineering, School of Engineering, Universitat Autònoma de Barcelona, Campus UAB, 08193, Bellaterra, Barcelona, Spain

[c] *MackGraphe-Mackenzie Institute for Research in Graphene and Nanotechnologies, Mackenzie Presbyterian University, Consolação Street 930, 01302-907, São Paulo, Brazil*

[d] *Department of Material Science, Universitat Autònoma de Barcelona, Campus UAB, 08193, Bellaterra, Barcelona, Spain*

[e] *Faculty of Bioscience and Technology for Food, Agriculture and Environment, University of Teramo, via Renato Balzarini 1, 64100 Teramo, Italy*

[f] *Catalan Institution for Research and Advanced Studies (ICREA), Passeig de Lluís Companys, 23, 08010, Barcelona, Spain*

* Co-corresponding authors: arben.merkoci@icn2.cat; giulio.rosati@icn2.cat, andrew.piper@icn2.cat

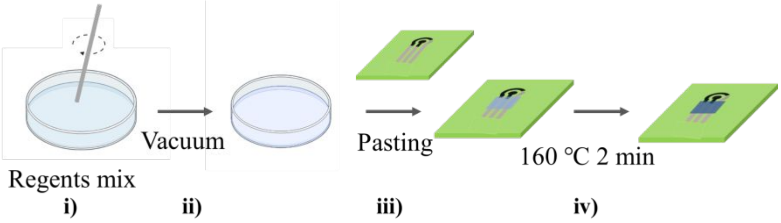

Figure S1 Schematic illustration of the insulation process using polydimethyl siloxane (PDMS). (i) Mix the base solution and curing agent in a 10:1 weight ratio; (ii) place in a vacuum chamber for 5 min to remove the bubbles generated during mixing; (iii) paste the PDMS over the desired area (between the electrodes and connection points); (iv) Cure at 160 °C for 2 min.

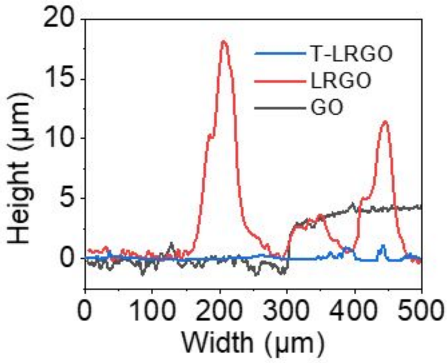

Figure S2. One-dimensional surface profiles of graphene oxide (GO), laser reduced graphene oxide (LRGO) and transferred laser reduced graphene oxide (T-LRGO). The data shows that the GO film is 3 - 4.5 μm thick. After laser induced reduction, the surface becomes much more heterogeneous but noticeably increases to a maximum height 18.2 μm in some areas, then decreases to 0.4 - 1.3 μm after transferring.

Table S1. XPS C 1s elemental composition in GO, LRGO and T-LRGO; C/O* means C/O ratio obtained by the relative peak area of C 1s and O 1s peak for the survey spectra.

GO			LRGO			T-LRGO		
C1s	Binding energy (eV)	Area percentage (%)	C1s	Binding energy (eV)	Area percentage (%)	C1s	Binding energy (eV)	Area percentage (%)
C-C	284.4	39.0	C-C	284.4	56.4	C-C	284.4	57.7
C-O	286.4	46.8	disorder	285.0	25.6	disorder	285.0	26.0
C=O	287.7	13.9	C-O	286.4	8.8	C-O	286.4	7.0
π-π*	291.2	0.3	π-π*	291.2	9.2	π-π*	291.2	9.4
C/O *	2.7		C/O *	22.0		C/O *	23.4	

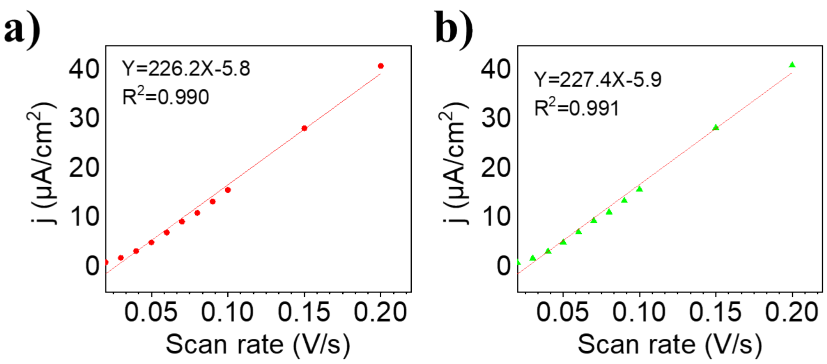

Figure S3. Current density at different scan rates obtained in a solution of 0.1 M $NaClO_4$ in ethanol with two T-LRGO electrodes, the dots represent the raw data and red lines are the fitted curves.

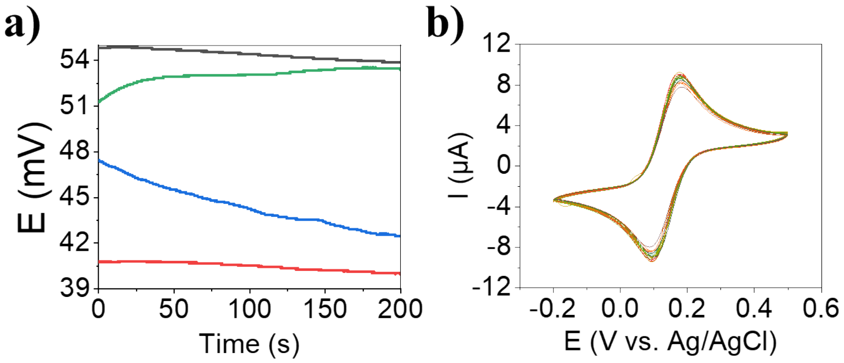

Figure S4 (a) Open circuit potential measurements of the as-prepared quasi-reference electrodes vs. a commercial Ag/AgCl reference electrode (b) CV measurements of PBASE functionalized electrodes (18 electrodes from 3 batches) in an aqueous solution of 0.5 mM $[Fe (CN)_6]^3$ / $[Fe (CN)_6]^4$ in 0.1 M KCl.

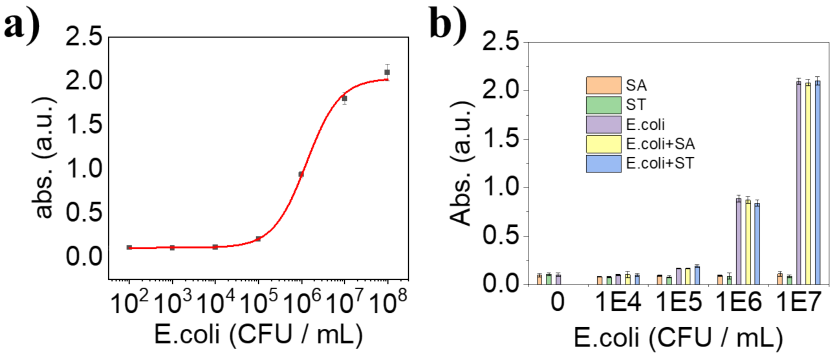

Figure S5 (a) calibration curve of *E. coli*. Obtained by a conventional ELISA (b) selectivity test by ELISA with cAb 2 µg/ml and dAb 0.33 µg/ml, N=3.

Table S2. 4-Parameter Logistic model fitting parameters for the standard optical ELISA and electrochemical ELISA.

Standard optical ELISA		Electrochemical ELISA	
Model	Logistic	Model	Logistic
Equation	y = A2 + (A1-A2)/(1 + (x/x0)^p)	Equation	y = A2 + (A1-A2)/(1 + (x/x0)^p)
Plot	abs.	Plot	Normalized response
A1	0.10967 ± 7.62939E-4	A1	-0.04481 ± 0.03554
A2	2.03463 ± 0.05476	A2	1.04113 ± 0.04522
x0	1300903.75685 ± 109381.62119	x0	133550.21188 ± 47258.68812
p	1.12889 ± 0.03679	p	0.37581 ± 0.05562
Reduced Chi-Sqr	0.62849	Reduced Chi-Sqr	0.00126
R-Square (COD)	0.99869	R-Square (COD)	0.99487
Adj. R-Square	0.9977	Adj. R-Square	0.99267

Table S3. Detailed timetable of electrode fabrication.

Steps	Time needed	Tools / Equipment	Number of substrates simultaneously processable in each step
Substrate cleaning	1 min	Isopropanol	~1
GO drop casting	5 min	micro- pipette	4~6
GO film forming	2 h	oven	~250
Laser reduction	10 min	Rayjet 50	~50
Stamp transferring	~1 min	mechanical press	1
Ag print	~10 min	Dimatix 2800	~20
Ag sinter	2 h	oven	~250
Doctor blading of PDMS	1 min	by hand	~4
PDMS solidification	2 min	oven	~250
Ag chlorination	3 min	micro- pipette	~3

One batch referes to 1 piece of substate on which 12 electrodes could be fabricated. The rate-limiting step in this process is the substrate treatment or stamp transferring process, which is limited to 48 electrode / 5 min. In ideal conditions and considering continuously working for 8 h; batches can be fabricated in parallel, allowing the fabrication of 2016 electrodes per day (8 h) by a single user.

Table S4 detailed material cost for the device fabrication.

Materials	Quantity	Price (€)	Electrode number per unit of materials	Price per device (€)
GO solution	1 L	300	24/5 mL	0.063
Polyester sheets	300 m^2	450	24/128 cm^2	0.001
Ag ink	50 mL	400	> 500/mL	0.016
PDMS	1 kg	700	60/g	0.012
cAb	1 mL	435	50/µL	0.009
dAb	250 µL	490	125/µL	0.016
Total price 0.1154 €				

Polyimide substrate, IM301451, DuPont™ Kapton® FPC, thickness 0.125 mm, area 0.61 m * 1 m=0.61 m^2 , ~700 €, ~1147 €/ m^2; GO solution, 5 mL liquid could cover a square area (8 cm × 8 cm), ~234 €/ m^2 .

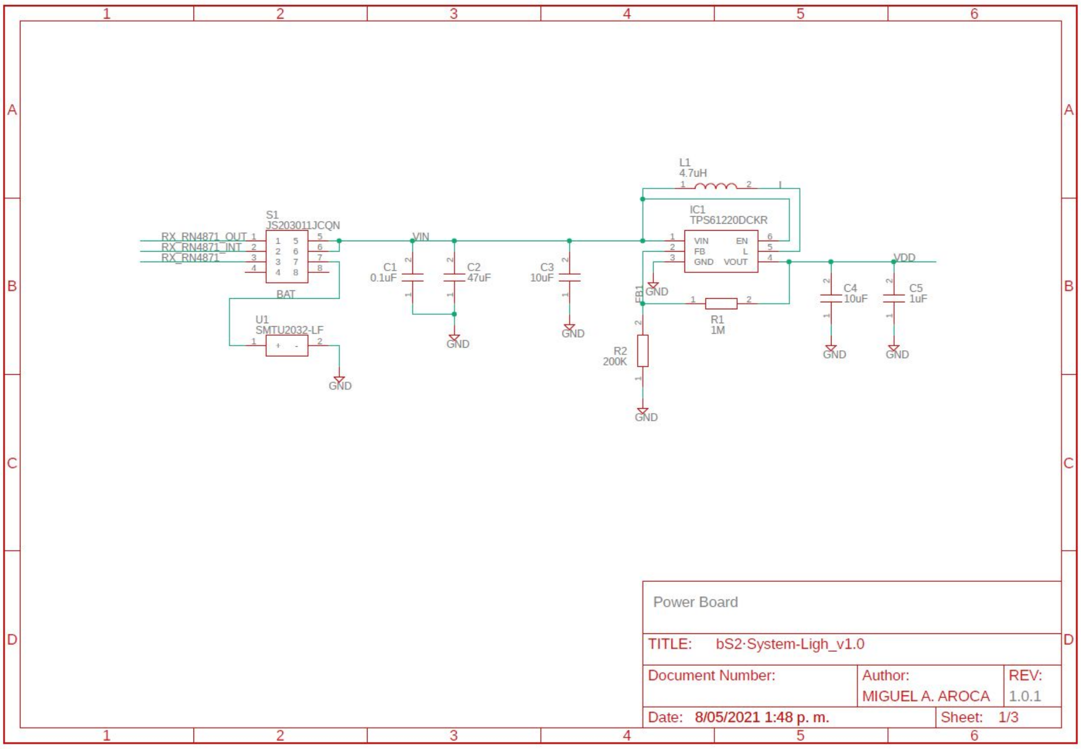
L1
4.7uH
IC1
TPS61220DCKR
S1
JS203011JCQN
RX_RN4871_OUT
RX_RN4871_INT
RX_RN4871
BAT
VIN
C1
0.1uF
C2
47uF
C3
10uF
U1
SMTU2032-LF
GND
FB1
R1
1M
R2
200K
C4
10uF
C5
1uF
VDD
Power Board
TITLE: bS2·System-Ligh_v1.0
Document Number:
Author:
MIGUEL A. AROCA
REV:
1.0.1
Date: 8/05/2021 1:48 p. m.
Sheet: 1/3

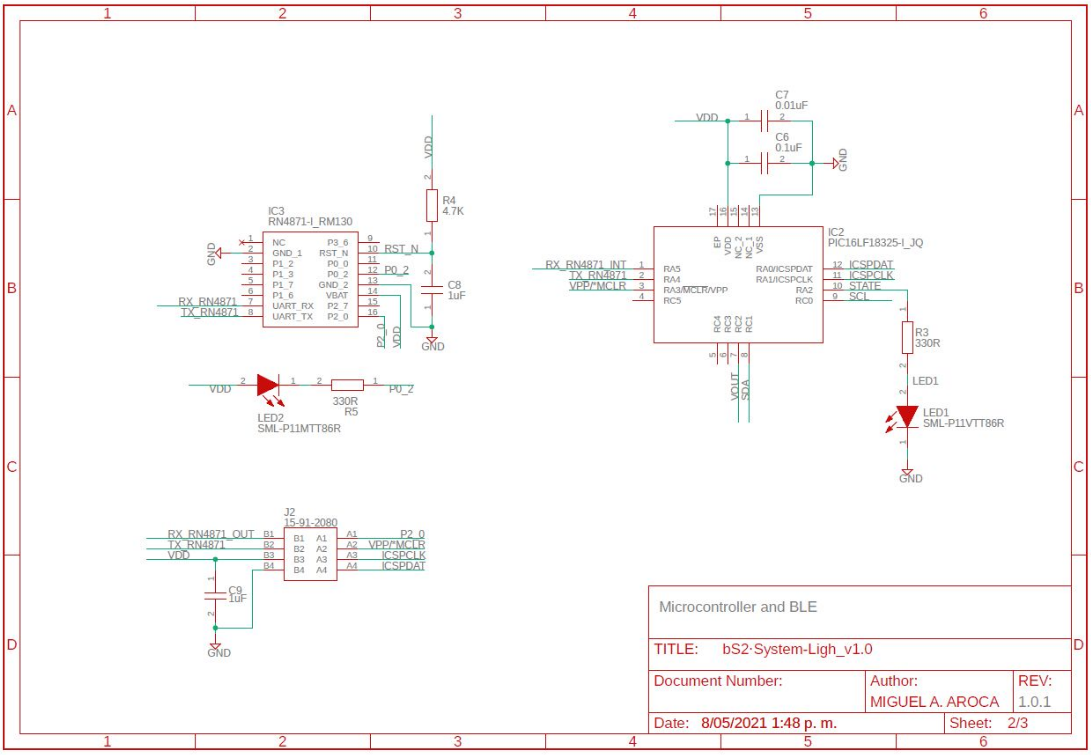
C7
0.01uF
C6
0.1uF
VDD
GND
R4
4.7K
IC3
RN4871-I_RM130
RST_N
P0_2
C8
1uF
RX_RN4871
TX_RN4871
P2_0
IC2
PIC16LF18325-I_JQ
RX_RN4871_INT
TX_RN4871
VPP/*MCLR
ICSPDAT
ICSPCLK
STATE
SCL
VOUT
SDA
R3
330R
LED1
SML-P11VTT86R
LED2
SML-P11MTT86R
330R
R5
J2
15-91-2080
RX_RN4871_OUT
C9
1uF
Microcontroller and BLE
TITLE: bS2·System-Ligh_v1.0
Document Number:
Author:
MIGUEL A. AROCA
REV:
1.0.1
Date: 8/05/2021 1:48 p. m.
Sheet: 2/3

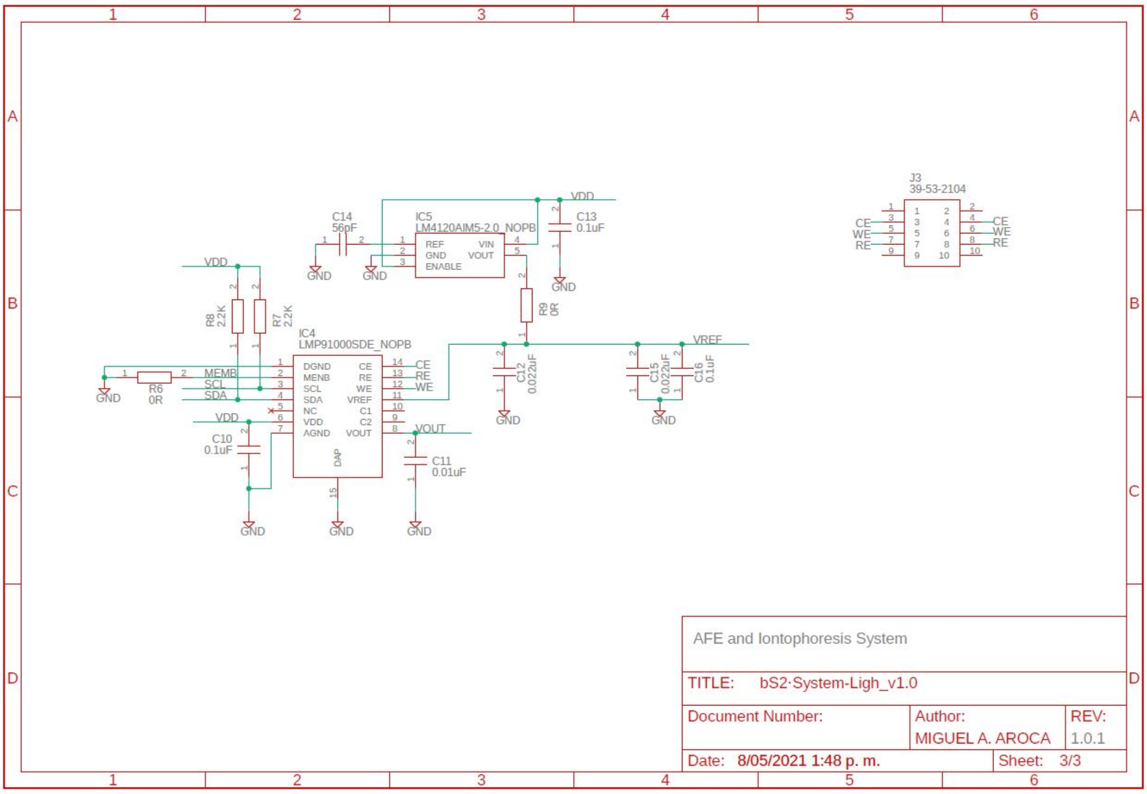

Scheme S1. Electric scheme of the wireless electrochemical measurement unit.

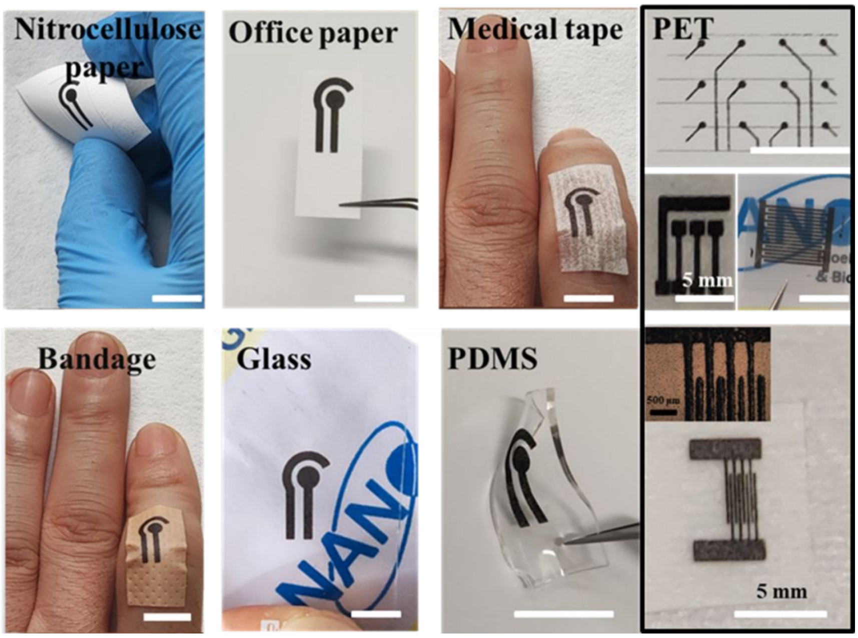

Figure S6 T-LRGO on various different substrates. The inset picture shows an interdigitated LRGO pattern before transferring. The scale bar is 1 cm unless labeled otherwise.
